# Endoscope-assisted resection of nonneoplastic space-occupying lesion in oral and maxillofacial areas

**DOI:** 10.1038/s41598-017-17226-z

**Published:** 2017-12-05

**Authors:** Yanan Li, Runqi Xue, Qingguo Lai, Bingbing Xu, Kuifeng Yuan, Xiaopeng Tang, Jiangbo Ci, Shaolong Sun, Zhichao Zhang

**Affiliations:** 1 Department of Oral and Maxillofacial Surgery, the Second Hospital of Shandong University, Jinan, China; 2 School of Medicine, Shandong University, Jinan, China; 3 School of Stomatology, Shandong University, Jinan, China

## Abstract

Endoscope-assisted oral and maxillofacial surgeries have been applied to the resection of tumors with minimal invasion and good cosmetic outcomes. However, with regard to endoscope-assisted resection of nonneoplastic space-occupying lesion (NSOL) in oral and maxillofacial areas which differ from tumors in treatment, there are no systematic reports. Therefore the advantages and limitations of the endoscopy-assisted approach (EAA) in resection of NSOL remain unclear. In this novel study we describe endoscope technique for resection of NSOL in face and submandibular areas and compare the feasibility and effectiveness of EAA with external approach (EA). Eleven patients underwent EAA and 20 patients underwent EA procedures. The perioperative and postoperative outcomes of the patients were evaluated. The resection of NSOL with EAA was completed successfully with a shorter hospitalization duration, less bleeding, a smaller incison and better satisfaction with appearance than with the EA procedure (*P < *0.01). Our study showed that endoscope-assisted resection of NSOL is technically safe, feasible and practicable. Good cosmetic results with minimal invasion can be achieved with this new technique and therefore this may be a promising new standard procedure in oral and maxillofacial areas.

## Introduction

Since endoscopy was introduced into the fields of craniomaxillofacial and facial plastic surgery in 1990^1^, minimally invasive endoscopic surgeries have been applied successfully to various procedures, including excision of tumors in the submandibular salivary gland, parotid gland and accessory parotid gland^2–11^, fixation of mandibular condyle fractures^12^ and repair of orbital wall fractures^13^. Many advantages were reported such as improved esthetic outcomes, fast recovery, less surgical complications and decreased tissue damage^2–10,14,15^.

However, the primary tumors of the parotid gland, accessory parotid gland and submandibular salivary gland are almost exclusively pleomorphic adenoma^14^. A pseudocapsule around the adenoma forms in the normal tissue resulting from the slow compression of expanding tumor to the circumferential tissue of the gland^16–20^. An avascular plane round the pseudocapsule is easily located and convenient for separating the tumors or glands during the endoscope-assisted surgery^21–23^. However intraoperative mucoid spillage of tumors can lead to recurrence of the disease due to incomplete removal of the pseudocapsule and satellitosis of the tumor^6,17,24,25^. Therefore partial excision around extracapsular tissue is recommended in the resection of pleomorphic adenoma^17–20,25^ and subcapsular dissection is not desirable in the endoscope-assisted resection of pleomorphic adenoma^7,8,14,18^. Given the poor operation field it is technically difficult to complete partial excision around extracapsular tissue in oral and maxillofacial areas^26^.

NSOL is very common in oral and maxillofacial areas and includes all kinds of cysts, vascular malformation and submaxillaritis. Subcapsular dissection may be sufficient for radical treatment of most of the NSOL, however in oral and maxillofacial surgery, operations on the face and submandibular areas of patients require a better cosmetic outcome than in other areas. Therefore, we believe that patients specifically suffering from NSOL in face and submandibular areas may significantly benefit from endoscope-assisted surgery.

To our best knowledge, there are no systematic reports with reference to endoscope-assisted resection of NSOL in oral and maxillofacial areas. In this novel study we describe our technique for resection of NSOL in face and submandibular areas and demonstrate its feasibility and effectiveness.

## Materials and Methods

### Subjects

Thirty-one patients with different NSOL underwent surgery between November 2012 and April 2016 in the Department of Oral and Maxillofacial surgery, at the Second Hospital of Shandong University. There were 11 male patients and 20 female patients, with ages ranging from 16 to 68 and a median age of 37 years. EAA were performed on 11 patients with NSOL in oral and maxillofacial areas, of which two patients were diagnosed as submandibular epidermoid cyst, two patients were diagnosed as IVM in front of parotid gland and seven patients suffered from submaxillaritis. The remaining twenty patients were treated with EA and included 5 submandibular epidermoid cysts, 3 IVM and 12 submaxillaritis. Computed tomography (CT) scan and/or magnetic resonance imaging (MRI) were used to evaluate the lesions of all the patients prior to surgery. All neoplastic space-occupying lesion or suspected cases of gland tumors were excluded from this study. This study was approved by the Ethics Committee of the Second Hospital of Shandong University. All patients received detailed information about the operative approach and signed informed consent prior to participating in this study. The patients with EAA were all informed that a conventional wide-open operation may be required if any surgical complications were encountered e.g. uncontrolled bleeding that could not be resolved by endoscope-assisted surgery, and that all the excised samples would be diagnosed by fast frozen pathology. If the patient was diagnosed with a tumor, a conventional open procedure would also be carried out. Statistical analysis of all of the data was performed using SPSS for Windows (SPSS Inc., Chicago, IL). Data are presented as mean values ±SD. For all analyses, the statistical differences were considered to be significant if *P* < 0.05.

### Surgical procedure

**Figure 1:**
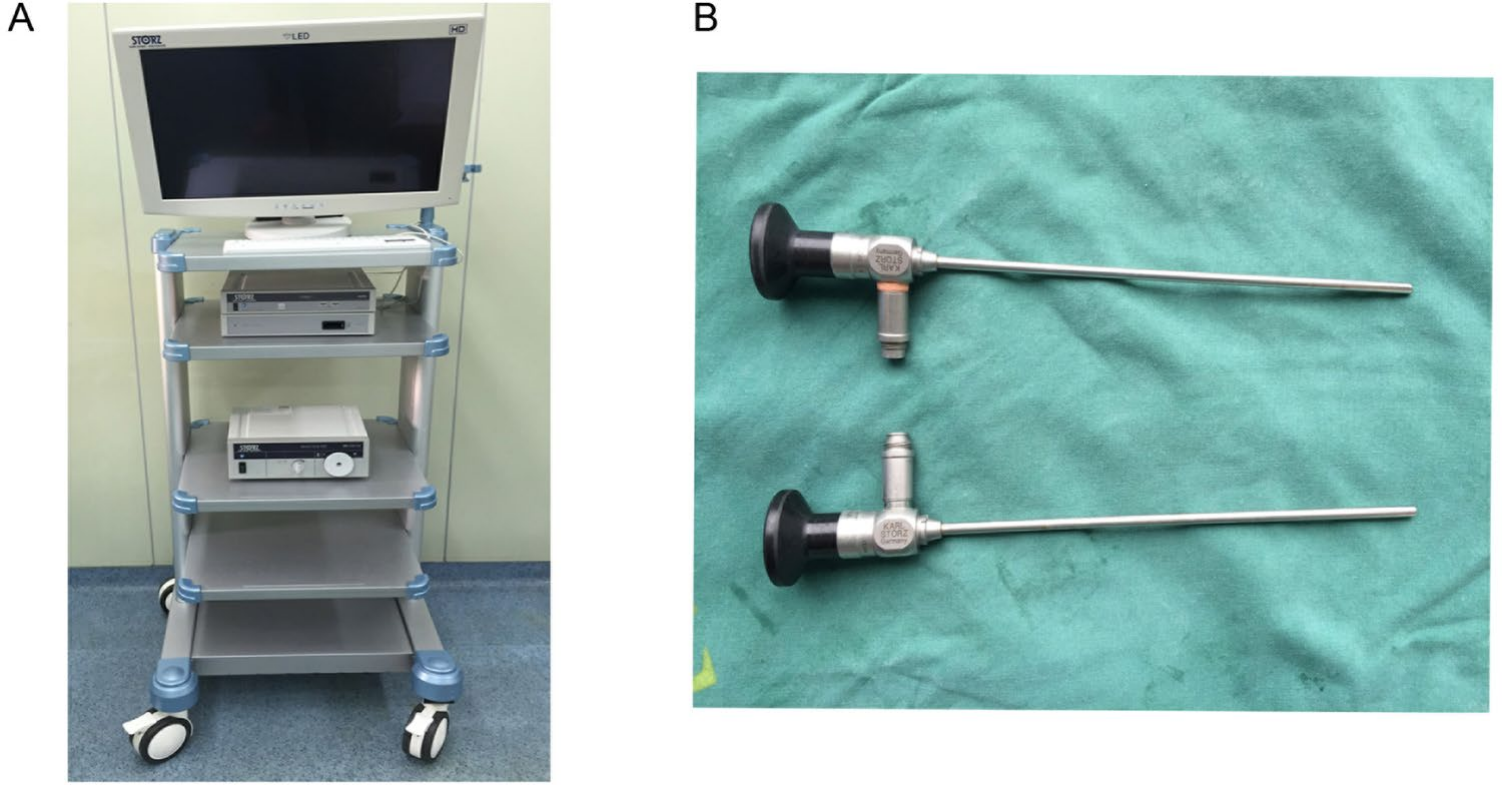
Images of the video camera system and endoscopes. (**A**) video camera system. (**B**) Image of a 30° 4-mm endoscope and a 0° 4-mm endoscope.

**Figure 2:**
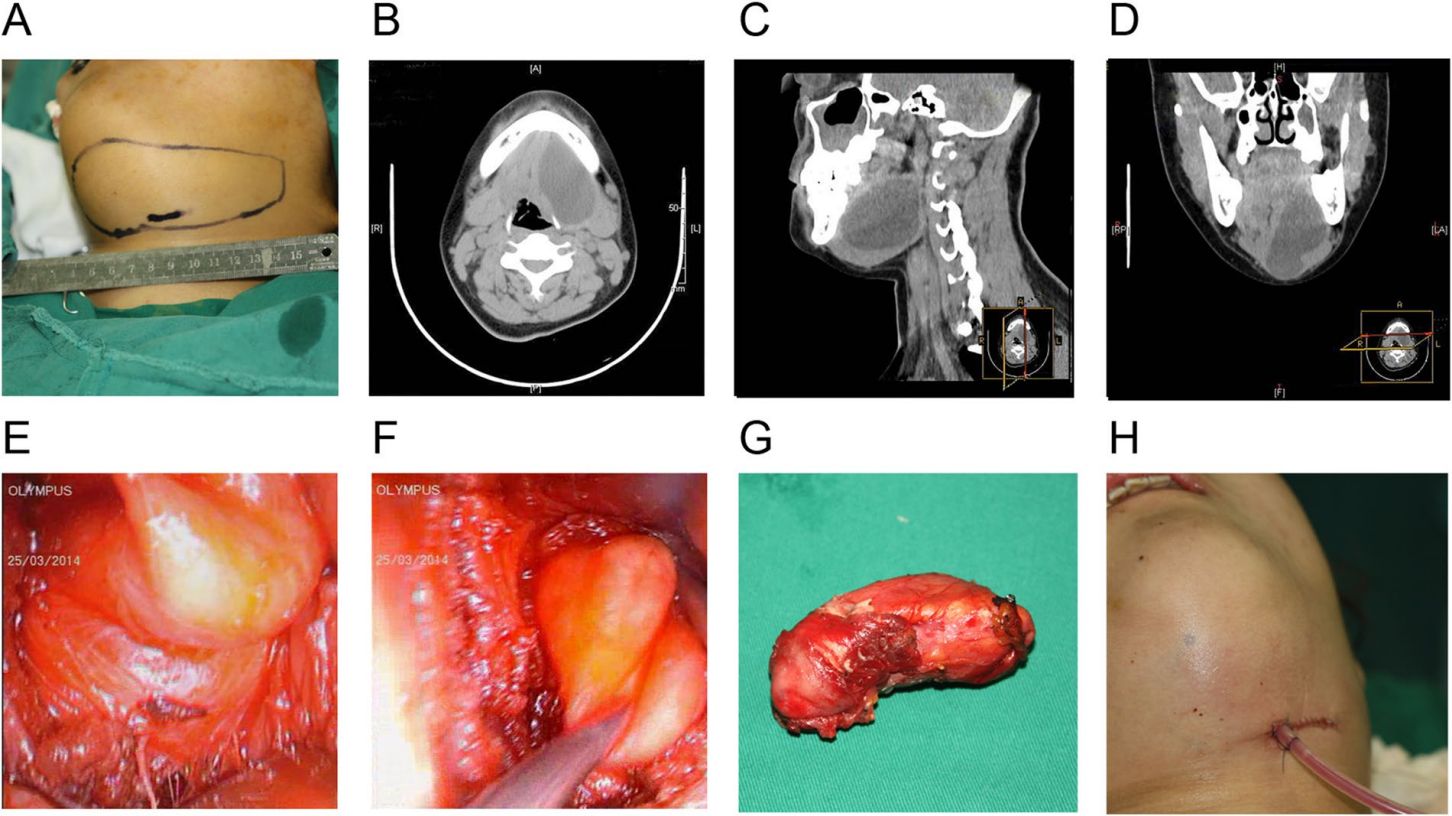
Endoscope-assisted resection of submandibular epidermoid cyst. (**A**) An incision planned on a 22-year-old girl diagnosed with epidermoid cyst. Plain scanning (**B**) and sagittal (**C**) and coronal (**D**) reconstruction were performed on the patient with epidermoid cyst. (**E**) The cyst was exposed under a clear working space. (**F**) Dissection of epidermoid cyst along an avascular plane around the cyst. (**G**) The resected sample of the epidermoid cyst. (**H**) A silastic drain was inserted deep in the wound after the operation.

Under general anesthesia, the patient’s neck was placed in the supine position with a pillow under the shoulder and extended, the head was then rotated to the opposite side of the lesion. A video camera system (Karl Stortz, Germany), a 30° 4-mm endoscope and a 0° 4-mm endoscope (Karl Stortz, Germany) were used (Fig. 1A,B). A 15- to 30-mm skin incision was made between the inferior and superior margin of the submandibular lesion, preferably in a natural cervical wrinkle over the middle of the protruding dome of the lesion (Fig. 2A). The CT examination results helped operators to evaluate the scope of the cyst in advance (Fig. 2B–D). The lesions (glands or cysts) were exposed after the incision of skin, subcutaneous tissue and platysma muscle. Then the dissection proceeded along an avascular plane round the pseudocapsule between the lesion and the adjacent tissue. This can decrease bleeding and get clear fields while minimizing the possibility of damaging the facial artery, vein and the marginal mandibular branch of the facial nerve. Special retractors, bipolar coagulation forceps and ultrasonic scalpels were also used in order to decrease the amount of bleeding and obtain clear fields (Fig. 2E). The first operation assistant was responsible for the correct positioning of the endoscope during the procedure. The main task of the second assistant was to provide maximum working space by lifting the skin flap away with two retractors. The facial artery and vein, Warthon’s duct and loop of the lingual nerve were identified and suture-ligated for the patients with submaxillaritis. As for epidermoid cysts the dissection along an avascular plane around the cyst was performed (Fig. 2F) and the cysts were removed through the surgical wound (Fig. 2G). The interior wounds were closed in layers with 5-0 absorbable sutures after irrigation, and the skin was stitched with 5-0 nylon sutures. A silastic drain was inserted deep in the wound and kept *in situ* for at least 2 days after the surgery (Fig. 2H).

**Figure 3:**
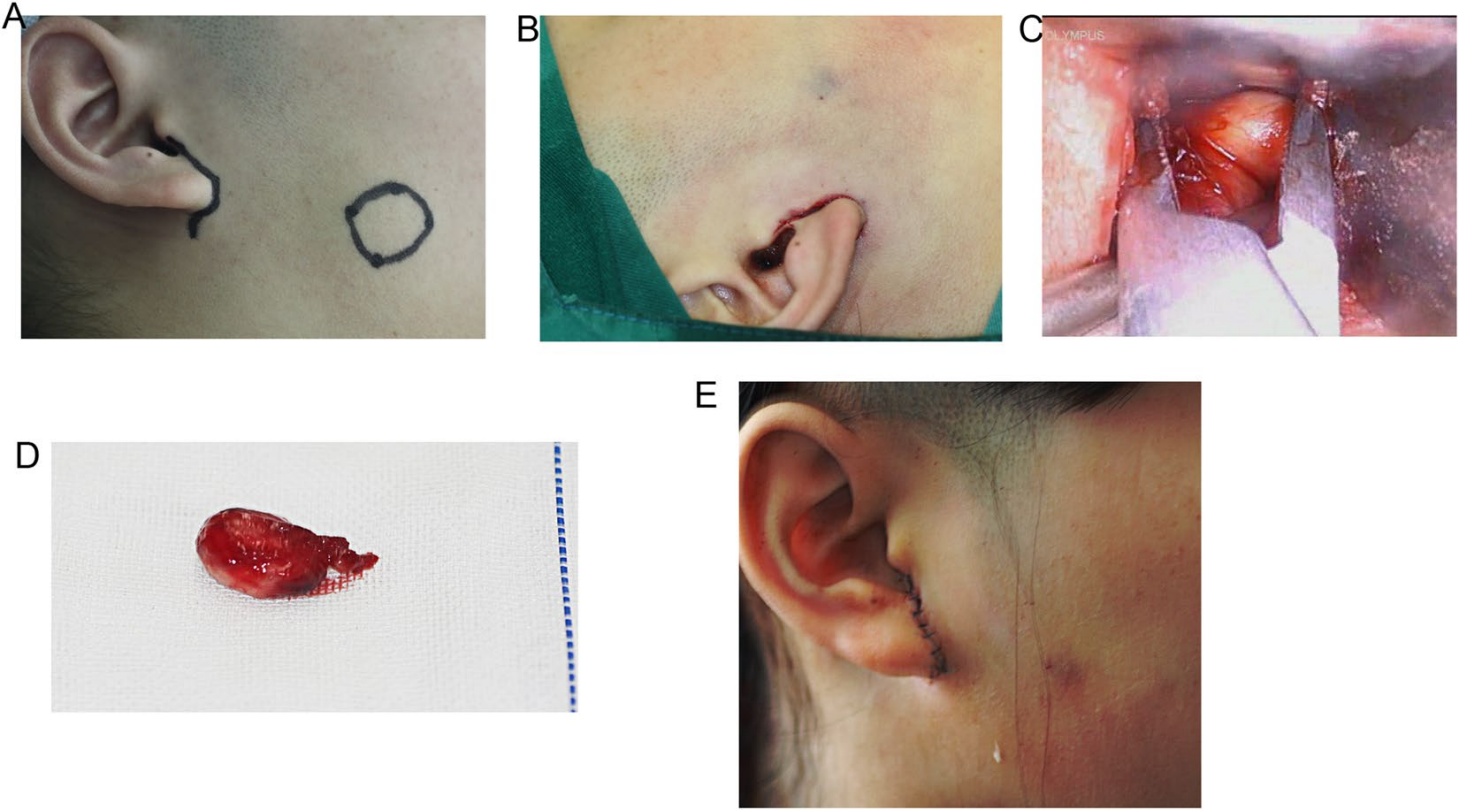
Endoscope-assisted resection of IVM in front of parotid gland. (**A**) A preauricular incision at the margin of the tragus was designed on a 16-year-old girl diagnosed as IVM in front of parotid gland. The scope of lesion was circled on the face preoperatively. (**B**) A small tragus incision approximately 2.5 cm long was performed. (**C**) From the tunnel the mass was clearly observed under endoscopic visualization. (**D**) The resected sample of IVM. (**E**) The image of incision after negative pressure drainage was removed two days after the operation.

In surgeries of the lesion in front of the parotid gland the same anesthesia and positioning of instruments were performed, with two assistants assisting the operator by positioning the endoscope and providing working space. The location of the lesion was marked on the face pre-operatively (Fig. 3A). A small tragus incision 2 cm to 2.5 cm long was made and did not extend beyond the inferior margin of the earlobe (Fig. 3B). An under skin tunnel was formed along the avascular plane around the surface of parotid gland capsule. The tunnel was extended to the mass with the same plane around the lesion, which was clearly observed under endoscopic visualization (Fig. 3C). The lesion was resected (Fig. 3D) with careful protection of nerves and other normal anatomic structures. Negative pressure drainage was removed two days after the operation (Fig. 3E).

### Postoperative outcome assessment

The perioperative and postoperative outcomes of the patients were assessed, including the operation time (from incision of the skin to stitching the skin), amount of operative bleeding, duration and volume of drainage, infection, overall hospitalization duration, length of incision, nerve injury and satisfaction score according to the cosmetic results and recurrence *in situ*. The volume of postoperative drainage was recorded every 24 hours, and the silastic tubes were removed once the drainage had reduced to less than 10 mL in a 24-hour period. The operative incision was defined as infection if it appeared obviously red and swollen with pus or required antibiotic treatment^27^. Postoperative pain one week after the operation was evaluated based on the visual analogue scale^27–29^. Patient satisfaction with cosmetic appearance at 3 weeks after surgery was also assessed using a visual analogue scale. The median follow-up durations ranged from 9–24 months after leaving hospital.

## Results

**Figure 4:**
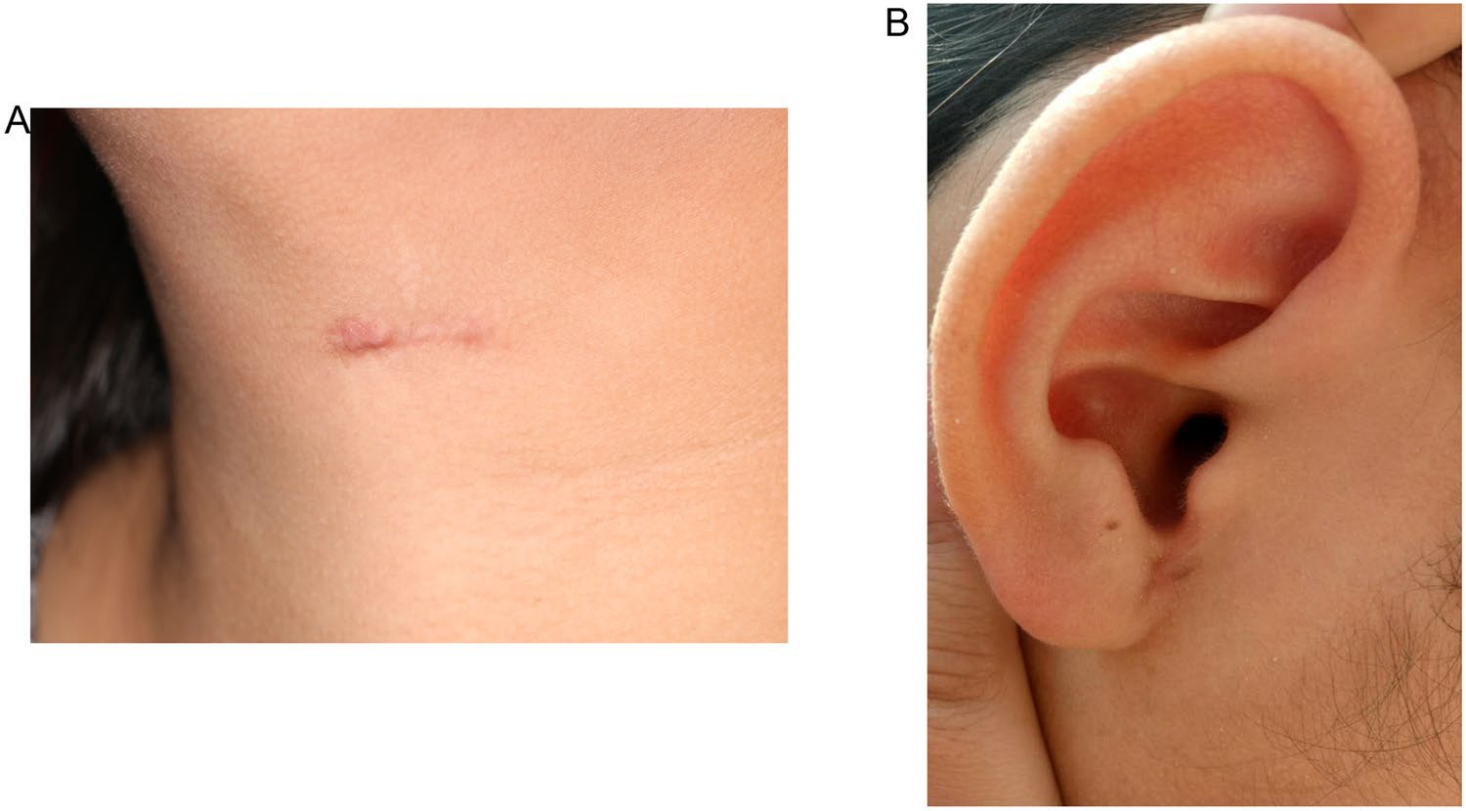
Cosmetic results after long term follow up. (**A**) After 24 months, the incision was esthetically pleasing even though this patient had scar diathesis. (**B**) After 10 months, the incision was no longer easily visible.

Clinical data of the patients and statistical analyses of all of the data are shown in Tables 1 and 2. All of the lesions in the 31 patients were resected completely and no patient suffered from complications such as infection, nerve injury or excessive bleeding. All eleven patients with EAA diagnosed by intraoperative frozen sections were treated by resection with the endoscopically assisted system and without having to revert to EA. There were significant statistical differences in the length of the wounds and the amount of intraoperative bleeding between the patients treated with EAA and those with EA (*P* < 0.001). However, the EAA procedure has a longer operation time compared with the EA procedure (90.64 ± 20.87 vs 52.05 ± 6.82 min, *P < *0.001). Comparison of postoperative drainage of the two methods showed no statistical difference, lso there was no significant difference in the postoperative pain score (*P* = 0.082). The average hospital stay of the patients with the EAA were shorter compared with the EA group (2.91 ± 0.74 vs 3.78 ± 0.70 day, *P* = 0.003). All the patients with EAA were significantly more satisfied with their cosmetic outcomes than those with the EA (*P* < 0.001). There were no postoperative complications, including recurrence, chronic infection, Frey syndrome or pain in any of the patients followed up for 9–24 months after leaving hospital and good cosmetic results were achieved in the long term follow up (Fig. 4A,B).

**Table 1:** Clinical data of the patients.

Operation method	Gender	Incision length,mm	Operation time,min	Intraoperative blood loss,ml	Amount of drainage,ml	Duration of drainage,hours	Postoperative pain score	Hospital stay, days	Satisfaction of appearance
EAA	M	20	96	12	41	47	3.5	3.5	9.0
EAA	F	30	120	30	74	72	4.0	3.0	8.5
EAA	F	20	87	23	49	50	2.5	2.0	9.0
EAA	F	25	90	18	44	50	5.0	3.0	8.5
EAA	F	25	112	21	60	53	4.5	3.0	8.0
EAA	M	25	78	17	48	46	5.5	3.0	9.5
EAA	M	20	107	27	51	51	4.0	4.0	10.0
EAA	F	25	103	26	38	44	3.0	2.0	9.5
EAA	F	20	91	28	35	41	3.5	2.5	8.5
EAA	M	15	50	11	35	43	2.5	2.0	9.0
EAA	F	20	63	22	53	49	4.0	4.0	8.5
EA	M	65	65	30	45	50	5.5	3.5	8.0
EA	F	70	55	50	42	48	5.0	5.5	6.5
EA	M	67	47	50	40	41	5.5	3.0	7.0
EA	F	79	45	55	53	51	3.5	4.0	6.5
EA	M	68	57	35	47	51	4.5	3.5	7.0
EA	F	65	47	60	53	52	3.5	4.0	5.0
EA	F	62	45	38	43	47	5.0	3.5	6.5
EA	F	80	50	47	65	68	4.5	3.5	7.5
EA	M	78	45	38	51	51	5.0	4.0	5.0
EA	F	76	60	28	39	50	5.5	5.0	6.5
EA	M	75	52	39	50	50	3.0	3.5	6.5
EA	F	69	48	46	45	68	3.5	3.0	5.0
EA	F	68	50	45	40	50	6.0	3.0	7.5
EA	F	67	40	36	41	49	3.5	3.5	7.0
EA	F	72	53	77	58	45	4.0	3.0	8.5
EA	F	74	60	25	50	55	4.5	5.0	7.0
EA	M	73	65	29	50	55	3.5	4.0	5.5
EA	F	78	55	25	53	48	4.0	4.0	7.5
EA	F	77	53	32	41	47	4.0	3.5	6.0
EA	M	74	49	78	43	46	5.0	3.5	5.5

**Table 2:** Statistical analyses of all of the data.

Parameter	EAA(n = 11)	EA(n = 20)	t/χ^2^	*P* value
Gender (F/M)	7/4	13/7	0.006	0.939
Age	35.73 ± 13.42	37.45 ± 12.11	−0.365	0.718
Incision length, mm	22.27 ± 4.10	71.85 ± 5.31	−26.792	<0.001
Operation time, min	90.64 ± 20.87	52.05 ± 6.82	5.959	<0.001
Intraoperative blood loss, ml	21.36 ± 6.33	43.15 ± 15.35	−5.547	<0.001
Amount of drainage, ml	48.00 ± 11.65	47.45 ± 6.85	0.166	0.869
Duration of drainage, hours	49.64 ± 8.27	51.00 ± 6.61	−0.540	0.594
Postoperative pain score	3.82 ± 0.96	4.42 ± 0.86	−1.804	0.082
Hospital stay, days	2.91 ± 0.74	3.78 ± 0.70	−3.246	0.003
Satisfaction of appearance	8.91 ± 0.58	6.58 ± 1.00	7.052	<0.001

## Discussion

Endoscope use was initially introduced to oral and maxillofacial surgery in laser lithotripsy of salivary gland stones by Königsberger R *et al*.^1^. Since then many surgeons have reported the application of endoscope use in oral and maxillofacial areas as it has several advantages over the conventional approach, including reduced tissue damage, a smaller wound, fewer wound-related complications, and minimal postoperative scarring^11,30–33^. Among the many advantages the good cosmetic result is the most satisfactory outcome to both patients and surgeons^14,34,35^. Other than in laparoscopic surgery, thoracoscopic surgery and endoscopic sinus surgery there was no natural anatomical space for endoscope-assisted surgery in oral and maxillofacial areas. Therefore the working space was formed artificially and was poor compared to natural space^14^. There is a general consensus that the tumors from parotid or sunmandibular gland should be partially excised around extracapsular tissue^18–20^. Given the poor operation space endoscope-assisted partial resection around extracapsular tissue requires a highly skilled technique, which prevents its popularization. Therefore, such operations are not yet a standard procedure in the head and neck region^14,36^

In this study, endoscope-assisted surgeries were performed on patients with NSOL in oral and maxillofacial areas with less intraoperative blood loss, shorter hospital stays and better cosmetic outcomes than those with EA. Subcapsular dissection was sufficient for radical treatment of all the patients without concerns about the incompletion of pseudocapsule and satellitosis of the tumor. All the endoscope-assisted surgeries were completed successfully. All patients received satisfactory cosmetic outcomes and no postoperative complications occurred during the months following surgery. In comparison resection of the submandibular lesions with EA is performed through a long cervical incision, which can result in a visible and troublesome scar. Additionally, the chance of damaging the marginal mandibular branch of facial nerve has been reported to be 1–7% in patients^37,38^. Endoscope-assisted technique allows for the manipulation of tissues in small spaces and provides improved access to lesions that might not be attainable by conventional surgical procedure. It provides excellent surgical exposure and achieves complete resection of the tumor with good haemostasis and minimum morbidity, while preserving the key structures^11^. Dissection along the avascular plane between the gland and the adjacent tissue with bipolar coagulation forceps or ultrasonic scalpels was easy because of the loose attachment between the surface of the gland and the connective tissue. The application of ultrasonic scalpels decreased the bleeding effectively, which provided a clear surgical field for operators. During endoscope-assisted operations the marginal mandibular branches of the facial nerve in all patients were well preserved because there was adequate distance between the dissection plane and the branch of nerve. Although sometimes the submandibular epidermoid cyst could be large the dissection along the surface of cyst was performed without difficulty. If the cyst was too big to go through the incision, suction of contents was performed to reduce the volume of the cyst. During the submandibular sialadenectomy the exposure of the loop of the lingual nerve, Wharton’s duct, and the accompanying vessels below the mylohyoid muscle plane was the key step. Every time we dissected this area the use of a 30° 4-mm endoscope and a 0° 4-mm endoscope were combined to secure a good surgical view as reported by other surgeons^4^. Some authors have reported that severe adhesion to the adjacent tissue existed in patients with submaxillaritis accompanying inflammation and as a result heavy bleeding took place during the procedure. Also, the lingual nerve was easily damaged in this situation due to the unclear working space^14^. Therefore, in patients with submaxillaritis an enhanced CT scan must be performed to exclude patients with inflammation^39^.

How to resect space-occupying lesions in front of parotid gland or near the accessory parotid gland is controversial clinically. The use of the Blair incision for the removal of lesion in front of the parotid gland results in a long and obvious scar, although the procedure provides adequate exposure of the operative field^40^. Intraoral incisions might get a better cosmetic result compared with extraoral incisions^41,42^, however there are many drawbacks with intraoral incisions such as the poor exposure of operation field, surgical wound infections and inconvenience for eating^43^ during recovery. In our study, a preauricular incision behind the ridge of the tragus was designed to extend downward along the crease between the ear and face to the interior margin of the earlobe. Complete resection of tumor with minimal disruption to the surrounding healthy tissue conformed to the principles of being minimally invasive^44^. This incision for EAA minimized the adverse effect of the scar visibility by using the natural structures around ear. For patients with an accessory tumor the smaller incision and longer distance from the lesion could increase the difficulty of the operation due to the challenge to realize the partial resection around the tumor^26^. However, resection of intermuscular vascular malformation in front of the parotid gland can be performed easily along the avascular plane around the pseudocapsule without fear of the incomplete removal of the pseudocapsule and satellitosis around the tumor.

There are still some limitations to the use of EAA. First, it is more time-consuming than EA due to the associated learning curve for perfecting the endoscope-assisted technique. Secondly, issues such as maintaining a clear a working space for the surgeon and how to adjust the endoscopes to the most suitable position need to be resolved. Thirdly, in cases of severe inflammatory adherences to the surrounding structures, the risk of vascular and neurological injury are increased.

## Conclusion

In this study we performed operations with EAA and EA on patients with NSOL in oral and maxillofacial areas and achieved complete resection of the NSOL. Patients with EAA achieved minimal invasion and good cosmetic results compared with those with EA. Endoscope-assisted resection of NSOL is more practicable for the beginner and so is promising as a standard procedure in the oral and maxillofacial areas.
